# Identification of potential transcriptional regulators of actinorhizal symbioses in *Casuarina glauca* and *Alnus glutinosa*

**DOI:** 10.1186/s12870-014-0342-z

**Published:** 2014-12-10

**Authors:** Issa Diédhiou, Alexandre Tromas, Maïmouna Cissoko, Krystelle Gray, Boris Parizot, Amandine Crabos, Nicole Alloisio, Pascale Fournier, Lorena Carro, Sergio Svistoonoff, Hassen Gherbi, Valérie Hocher, Diaga Diouf, Laurent Laplaze, Antony Champion

**Affiliations:** Laboratoire Mixte International Adaptation des Plantes et microorganismes associés aux Stress Environnementaux, Centre de Recherche de Bel Air, BP 1386 CP 18524 Dakar, Sénégal; Laboratoire Commun de Microbiologie IRD/ISRA/UCAD, Centre de Recherche de Bel Air, Dakar, Sénégal; Laboratoire Campus de Biotechnologies Végétales, Département de Biologie Végétale, Faculté des Sciences et Techniques, Université Cheikh Anta Diop, Dakar, BP 5005 Dakar-Fann Sénégal; Institut de Recherche pour le Développement (IRD), UMR DIADE, Equipe Rhizogenèse, Montpellier, France; Department of Plant Systems Biology, VIB, Ghent, Belgium; Department of Plant Biotechnology and Bioinformatics, Ghent University, Ghent, Belgium; Université Lyon 1, Université de Lyon, CNRS, Ecologie Microbienne, UMR 5557, Villeurbanne, 69622 Cedex France

**Keywords:** Transcription factors, Symbiosis, Actinorhizal plants, Signaling pathway, C_2_H_2_ transcription factors

## Abstract

**Background:**

Trees belonging to the *Casuarinaceae* and *Betulaceae* families play an important ecological role and are useful tools in forestry for degraded land rehabilitation and reforestation. These functions are linked to their capacity to establish symbiotic relationships with a nitrogen-fixing soil bacterium of the genus *Frankia*. However, the molecular mechanisms controlling the establishment of these symbioses are poorly understood. The aim of this work was to identify potential transcription factors involved in the establishment and functioning of actinorhizal symbioses.

**Results:**

We identified 202 putative transcription factors by *in silico* analysis in 40 families in *Casuarina glauca* (*Casuarinaceae*) and 195 in 35 families in *Alnus glutinosa* (*Betulaceae*) EST databases. Based on published transcriptome datasets and quantitative PCR analysis, we found that 39% and 26% of these transcription factors were regulated during *C. glauca* and *A. glutinosa-Frankia* interactions, respectively. Phylogenetic studies confirmed the presence of common key transcription factors such as NSP, NF-YA and ERN-related proteins involved in nodule formation in legumes, which confirm the existence of a common symbiosis signaling pathway in nitrogen-fixing root nodule symbioses. We also identified an actinorhizal-specific transcription factor belonging to the zinc finger C1-2i subfamily we named *CgZF1* in *C. glauca* and *AgZF1* in *A. glutinosa*.

**Conclusions:**

We identified putative nodulation-associated transcription factors with particular emphasis on members of the GRAS, NF-YA, ERF and C_2_H_2_ families. Interestingly, comparison of the non-legume and legume TF with signaling elements from actinorhizal species revealed a new subgroup of nodule-specific C_2_H_2_ TF that could be specifically involved in actinorhizal symbioses. *In silico* identification, transcript analysis, and phylogeny reconstruction of transcription factor families paves the way for the study of specific molecular regulation of symbiosis in response to *Frankia* infection.

**Electronic supplementary material:**

The online version of this article (doi:10.1186/s12870-014-0342-z) contains supplementary material, which is available to authorized users.

## Background

Nitrogen is one of the most limiting nutrients for plant growth despite its abundance in the atmosphere. It can only be absorbed by plants as NH_4_^+^ or NO_3_^−^. However, dinitrogen can be reduced by some diazotrophic soil microorganisms, some of which are able to associate with specific plant families. Some of these mutualistic associations lead to the accommodation of bacteria within plant cells in specialized root structures called root nodules. Root nodule symbioses are found in a limited number of plants belonging to the legume, *Cannabaceae* (*Parasponia*) and actinorhizal plants. Actinorhizal plants belong to eight angiosperm families that can form symbioses with a filamentous soil bacterium called *Frankia*. Despite their ecological importance and recent advances in knowledge, the molecular bases of the formation and functioning of actinorhizal symbioses are still poorly understood [1-4]. Recently, transcriptomic analyses in three actinorhizal plants, *C. glauca*, *A. glutinosa* and *Datisca glomerata* [5,6] led to the discovery and characterization of several genes preferentially expressed in actinorhizal nodules following inoculation with *Frankia*. This global analysis of gene expression revealed that genes of the common symbiotic pathway (SYM) composed of signaling elements required for both root nodule and arbuscular mycorrhizal (AM) symbioses were also conserved in actinorhizal plants [5,6]. Furthermore, comparative transcriptome analysis of genes expressed during AM, rhizobial and actinorhizal symbioses suggests the existence of a core set of genes induced in these three endosymbioses [7].

In the last decade, genetic studies on legume symbiosis of the model species (*Lotus japonicus* and *Medicago truncatula*) have elucidated the role of transcription factors (TF) at different stages of nodule formation [8]. The first TF described as playing a role in *Lotus japonicus* nodulation was the *NIN* (nodule inception) gene for which mutation leads to inhibition of infection and primordia formation [9]. *NIN* is a transcription factor that is induced during the early stages of nodule organogenesis [10] and is involved in many nodule formation processes [11]. In addition, *NIN* orthologs have been identified in pea, soybean and *M. truncatula*, where they act downstream of the common *SYM* genes [12]. Recently, it was demonstrated that in *L. japonicus*, *NIN* regulates cortical cell division by targeting two nuclear factors, *LjNF*-*YA1* and *LjNF*-*YB1,* that are essential for root nodule organogenesis [13]. Furthermore, it was demonstrated that the TF CYCLOPS transactivates *NIN* expression in a phosphorylation-dependent manner leading to root nodule development [14]. *NSP1* and *NSP2* (nodulation signaling pathway) genes coding for GRAS TF are also specifically involved in the rhizobia-legume symbiosis [15-17], and recent studies suggest their involvement in the arbuscular mycorrhizal symbiosis [18,19]. NSP1/NSP2 forms an heterodimer and activates the *ERN1* (ethylene response factor) TF required for nodulation by binding to the AT rich region of the promoter, which in turn stimulates the expression of the *ENOD11* gene expressed during the pre-infection process [17]. In *Medicago truncatula*, MtNF-YA1 and MtNF-YA2 also control *ENOD11* expression through direct *MtERN1* activation [20]. Recently, *MtRSD* (regulator of symbiosome differentiation), a Cysteine-2/Histidine-2 (C_2_H_2_) TF, was shown to promote differentiation of bacteria into nitrogen fixing bacteroids [21]. Taken together, these data reveal that specific TF orchestrate plant infection and nodule organogenesis in legumes.

The objective of this study was to identify the TF that regulate the expression of genes involved in the *C. glauca- /A. glutinosa-Frankia* actinorhizal symbioses. Among 14,000 unigenes expressed in roots and nodules of each of the two species, we identified 202 and 195 TF distributed in 40 and 35 families in *C. glauca* and *A. glutinosa,* respectively. A global analysis of the expression profile of these genes was conducted to identify up- and down-regulated TF encoding genes in nodule versus root, as well as nodule-specific TF. The expression level of several *C. glauca* and *A. glutinosa* TF was confirmed by quantitative PCR. Phylogenetic analyses performed in model legumes, species related to actinorhizal plants, and the actinorhizal plants *C. glauca* and *A. glutinosa* enabled us to identify ZF1 (zinc finger 1)-related transcription factors as potential specific regulators of actinorhizal symbioses.

## Results

### Identification of *C. glauca* and *A. glutinosa* transcription factors

To identify transcription factors in actinorhizal plants, tBLASTn searches of the *C. glauca* and *A. glutinosa* unigene databases were performed using the DNA-binding domain from the TF database of *Arabidopsis thaliana* as query sequences. These databases contain 14,327 unigenes for *A. glutinosa* and 14,868 unigenes for *C. glauca* [5]. BLAST analysis of the two unigene sets revealed 405 and 358 genes possibly encoding TF in *C. glauca* and *A. glutinosa,* respectively. To remove false positives, tBLASTx was performed to check trans-species sequence homologies between Arabidopsis genes and actinorhizal sequences with an e-value cut-off of 1e^−10^. Using this approach, we narrowed it down to 202 and 195 potential transcription factors distributed in 40 and 35 families in *C. glauca* and *A. glutinosa,* respectively (Additional files 1 and 2). No potential members were identified for thirteen families including M-type, E2F/DP, and GeBP in *C. glauca* and *A. glutinosa* unigene databases. Each predicted *C. glauca* and *A. glutinosa* TF gene was given an arbitrary number. Additional files 1 and 2 list each predicted gene for both species, together with the accession numbers of all unigenes, the closest Arabidopsis TF, and detailed BLAST information. Among TF families, the MYB superfamily and the ERF family were the largest, totaling 39 TF for each species. The third largest family was the C_2_H_2_ family, with 18 members in *C. glauca* and 20 in *A. glutinosa*, followed by the WRKY, NAC and bHLH families. The remaining families were represented by 1 to 19 members (Table 1).

**Table 1:** **Classification of putative transcription factor of** ***Casuarina glauca*** **and** ***Alnus glutinosa*** **into families**

**TF family**	***Casuarina glauca***	***Alnus glutinosa***	**Total TF**	**Domain description**
AP2	3	2	5	AP2 domain
ARF	6	4	10	Auxin response factor
ARR-B	1		1	Response regulator contain MYB-like DNA binding domain ARRM (type B)
B3	1		1	AP2 like transcriptional factor
BBR/BPC	1		1	Basic pentacysteine
BES1	1		1	BRI1-EMS-SUPPRESSOR 1
bHLH	8	15	23	The basic/helix-loop-helix proteins
bZIP	8	10	18	Basic Leu zipper (bZIP) TF
C2H2	18	20	38	Zinc finger, C2H2 type
C3H	2	3	5	Zn-finger, C-x8-C-x5-C-x3-H type
CAMTA	1		1	Calmodulin binding transcription factors
Co-like	2	2	4	CONSTANS TF,defined by zinc finger N-terminal and CCT domain C-terminal
DBB	4	3	7	Double B-box zinc finger
Dof	5	1	6	DNA binding with one finger
EIL	1	2	3	Ethylene insensitive 3
ERF	15	24	39	Ethylene response factor
FAR1	2	1	3	Far-red impaired responsive directly active transcription of FHY1 and FHL
G2-like	7		7	Homeodomain-like, GLK proteins belonging GARP superfamily
GATA	1	3	4	Zinc-finger Animal contain two C-x2-Cx17-C-x2-C domains type-IV
GRAS	11	8	19	SCARECROW (SCR), SHORTROOT (SHR) and DELLA domains
GRF		1	1	GROWTH-REGULATING FACTOR
HB-other	1	1	2	Homeobox domain
HD-ZIP	3	6	9	HD-ZIP protein, N terminus
HSF	1	4	5	Heat stress transcription factors, DNA binding C-terminal domains
LBD		1	1	LOB domain
LSD	2	3	5	zinc finger domains, CxxCxRxxLMYxxGASxVxCxxC type
MIKC	14	5	19	MADS-box, MIKC type
MYB	20	19	39	MYB DNA-binding domain
MYB-related	3	3	6	N-terminal myb-domain
NAC	14	15	29	No apical meristem (NAM), N-terminal DNA-binding domain and a C-terminal domain
NF-YA	2	4	6	subunit NF-YA, Gln(Q)- and Ser/Thr(S/T)-rich NH2 termini, and a DNA-binding domain
NF-YB	1	4	5	NF-Y TF, subunit NF-YB related H2B histones, DNA binding domain
NF-YC	4	5	9	NF-Y TF, subunit NF-YC related H2A histones, DNA binding domain
Nin-like	2		2	Plant regulator RWP-RK
RAV		1	1	B3 domain, DNA binding domain and single AP2/ERF domain
S1Fa-like	1	1	2	S1F binding site, NLS and a putative DNA binding helix
SBP	1		1	Two zinc-binding sites, Cys3HisCys2HisCys or Cys6HisCys sequence motif
SRS		1	1	Zn-finger, LRP1 type
TALE	5	3	8	Homeodomain
TCP	2	2	4	TCP TF
Trihelix	1	2	3	DNA-binding domain, GT factor
WOX	1		1	Homeobox domain, WOX class
WRKY	15	15	30	DNA-binding WRKY
Whirly		1	1	DNA-binding, Defense response
ZF-HD	1		1	Homeobox domain, ZF-HD class
**Total TF**	**202**	**195**	**397**	
**Total families**	**40**	**35**	**45**	

TF = Transcription factor.

### Expression profile of the putative actinorhizal transcription factors

**Figure 1:**
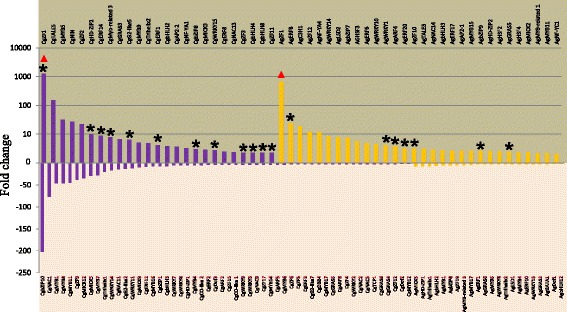
**Expression profiles of potential transcription factors in** ***Casuarina glauca*** **and** ***Alnus glutinosa*** **nodules compared to non-inoculated roots.** Purple and orange bars represent putative transcriptional regulators from *C. glauca* and *A. glutinosa* respectively. Bars represent fold change in nodules compared to non-inoculated roots (Fold change or FC ≥ 2 or ≤ −2 and p-value < 0.01). Transcription factors induced and repressed in *C. glauca* and *A. glutinosa* nodules are highlighted in tanned brown and light pink respectively. Astericks identify the genes confirmed by Q-PCR. Red triangles identify the transcription factors (*CgZF1* and *AgZF1*) most strongly induced in the nodules.

Microarray data was retrieved from previous published studies [5,7]. First, we used a simple comparative transcriptomics tool called *Casuarina* Transcriptome Compendium (CTC) to compare the microarray data and generate expression profiles in different conditions. Using CTC, we identified 54 repressed transcription factors and 25 induced in *C. glauca* nodules compared to non-inoculated roots with a nodule/root fold change ≥ 2 or ≤ −2 and a *p-value* ≤ 0.01 (Figure 1 and Additional file 3). The C_2_H_2_ family was the most frequently represented in the up-regulated class followed by the ERF and bHLH families (Additional file 4). Induction of 11 transcription factors in *C. glauca* nodules was confirmed by quantitative PCR, which also revealed similar induction values than microarray data (Additional file 5). Induction of *CgZF1* was validated by semi-quantitative PCR because its expression was not detectable in the non-inoculated roots needed to calibrate Q-PCR analysis (Additional file 6).

Similarly, we combined available microarray data in *A. glutinosa* to generate an *Alnus* Transcriptome Compendium (ATC). This allowed us to identify 30 putative transcription factors induced and 22 others repressed in *A. glutinosa* nodules compared to non-inoculated roots (Figure 1 and Additional file 7). Similar to the results observed in *C. glauca* nodules most MYB and WRKY were down-regulated while C_2_H_2_ were induced in nodules, (Additional file 8). Induction in *A. glutinosa* nodules of seven transcription factors was also confirmed by quantitative PCR (Additional file 5).

### Comparison of TF gene expression in AM and actinorhizal symbioses

**Figure 2:**
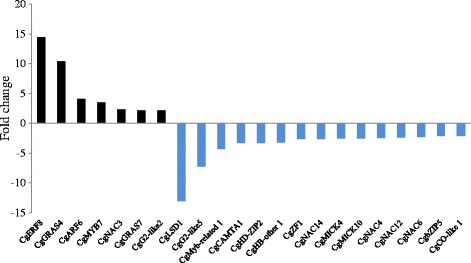
**Expression profiles of potential transcription factors in** ***C. glauca*** **mycorrhizal roots compared to non-inoculated roots.** Bars represent fold change in mycorrhizal roots compared to non-inoculated roots (Fold change or FC ≥ 2 or ≤ −2 and p-value < 0.01). Black and blue bars represent up and down-regulated transcriptional regulators in *C.glauca* mycorrhizal roots. Transcription factors induced and repressed in *C. glauca* mycorrhizal roots are highlighted in tanned brown and light pink respectively.

This first set of expression data prompted us to investigate TF gene expression during AM symbiosis in *C. glauca*. Mycorrhized roots data were retrieved from a recent publication [7]. Transcriptomic data highlighted 15 downregulated and 7 up-regulated transcription factors in *C. glauca* mycorrhized roots (Figure 2 and Additional file 9). NAC and GRAS families had the highest number of transcription factors regulated in mycorrhized roots (Additional file 4). Comparing expression in nodules and mycorrhized roots led to the identification of TFs induced during root nodule symbioses but not during AM. Only *CgZF1*, *CgHD-ZIP2* and *CgG2-like5* respectively belonging to C_2_H_2_, HD-ZIP and G2-like families were significantly induced in response to infection by *Frankia* and not induced in response to AM fungi (Figures 1 and 2). Even though root nodule symbioses are hypothesized to originate from AM, comparative analyses showed that only one transcription factor named *CgERF8* (Nod up/Myc up) was induced both in nodules and mycorrhized roots of *C. glauca*. Interestingly, *CgERF8* was identified as being related to ERN genes from *M. truncatula* known to be induced by *Rhizobium* and AM [5].

### GRAS, NF-YA and ERF transcription factor families in actinorhizal species

In legumes, several TFs that play key roles in the transduction of bacterial Nod factors have been identified. These include in particular members of the GRAS, NF-YA and ERF families (*i.e.* NSP1, NSP2, NF-YA1, NF-YA2, ERN1, ERN2 and ERN3) [15,20,22]. Therefore we first focused on these families. We screened extra databases using the tBLASTn algorithm in order to identify GRAS, NF-YA and ERF TFs in a third actinorhizal species, *D. glomerata* [6], relatives of actinorhizal plants in the Rosales and Cucurbitales orders, in legumes (http://www.phytozome.net/search.php?show=blast&method=Node_rosales-cucurbitales) and in Arabidopsis (TAIR).

**Figure 3:**
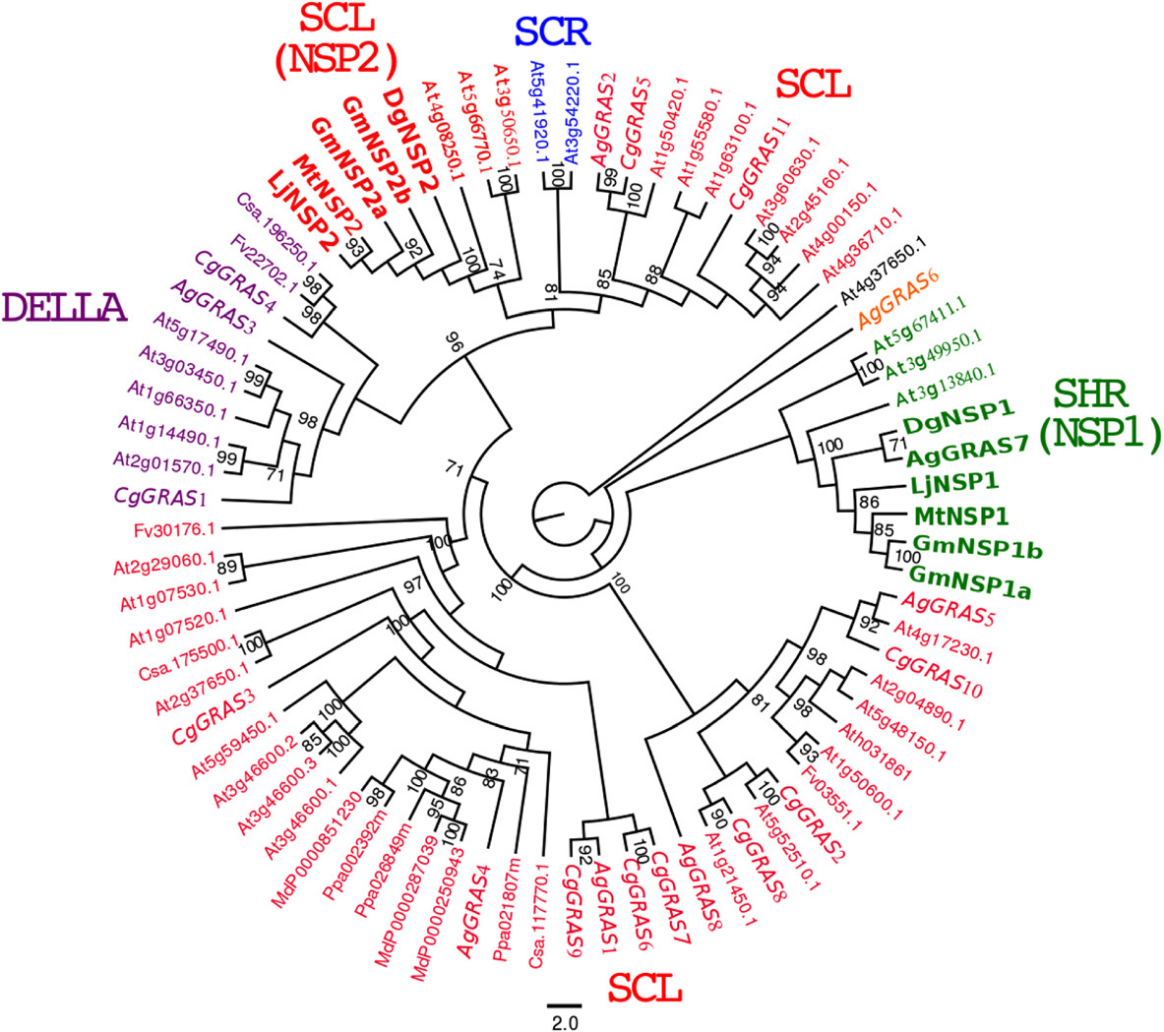
**Phylogenetic tree of the GRAS protein family.** Three GRAS proteins from *C. sativus*, *P. persica*, *M. domestica* and *F. vesca* each were retrieved using a BLAST-P search performed in phytozome and two sequences DgNSP1 (comp755_c1_seq1) and DgNSP2 (comp1841_c1_seq1) of *D. glomerata* (https://fido.nsc.liu.se/) [6]. We also include 36 sequences available in the genomes of *A. thaliana*, 2 from *M. truncatula,* 2 from *L. japonicus,* 4 from *G.max*, 11 from *C. glauca* and 8 from *A. glutinosa*. In italic, transcription factors *C.glauca* and *A.glutinosa*. The GRAS family comprises 4 subfamilies referred to as SCL, SCR, SHR and DELLA. NSP1 and 2 belong to SHR and SCL subfamilies respectively. *AgGRAS7* is closely related to *DgNSP1* (green fat letters). No sequence from *C. glauca* is present in the NSP1 and NSP 2 groups (red fat letters). The tree was rooted with *A. thaliana* sequence AT3G37650.1. One hundred bootstrap replications were used to evaluate statistical support for branches. Branches with less than 70% bootstrap support were collapsed.

The data was used to construct a phylogenetic tree of the GRAS protein family based on the alignment of the complete protein sequences using the maximum likelihood method. Eleven complete *C. glauca* sequences and eight *A. glutinosa* were collected from respective datasets. Based on the phylogenetic analyses, four distinct subfamilies were defined: SCARECROW-like (SCL), SCARECROW (SCR), SHORTROOT (SHR) and DELLA. The tree topology resembled the one found both in rice and Arabidopsis [23,24]. No sequence from *C. glauca* was close to NSP1 and NSP2. The same result was found for Rosales and Cucurbitales. However, two sequences from *A. glutinosa* and *D. glomerata* named AgGRAS7 (AG-J07f_002_D03) and DgNSP1 respectively were identified in the NSP1 group (Figure 3).

**Figure 4:**
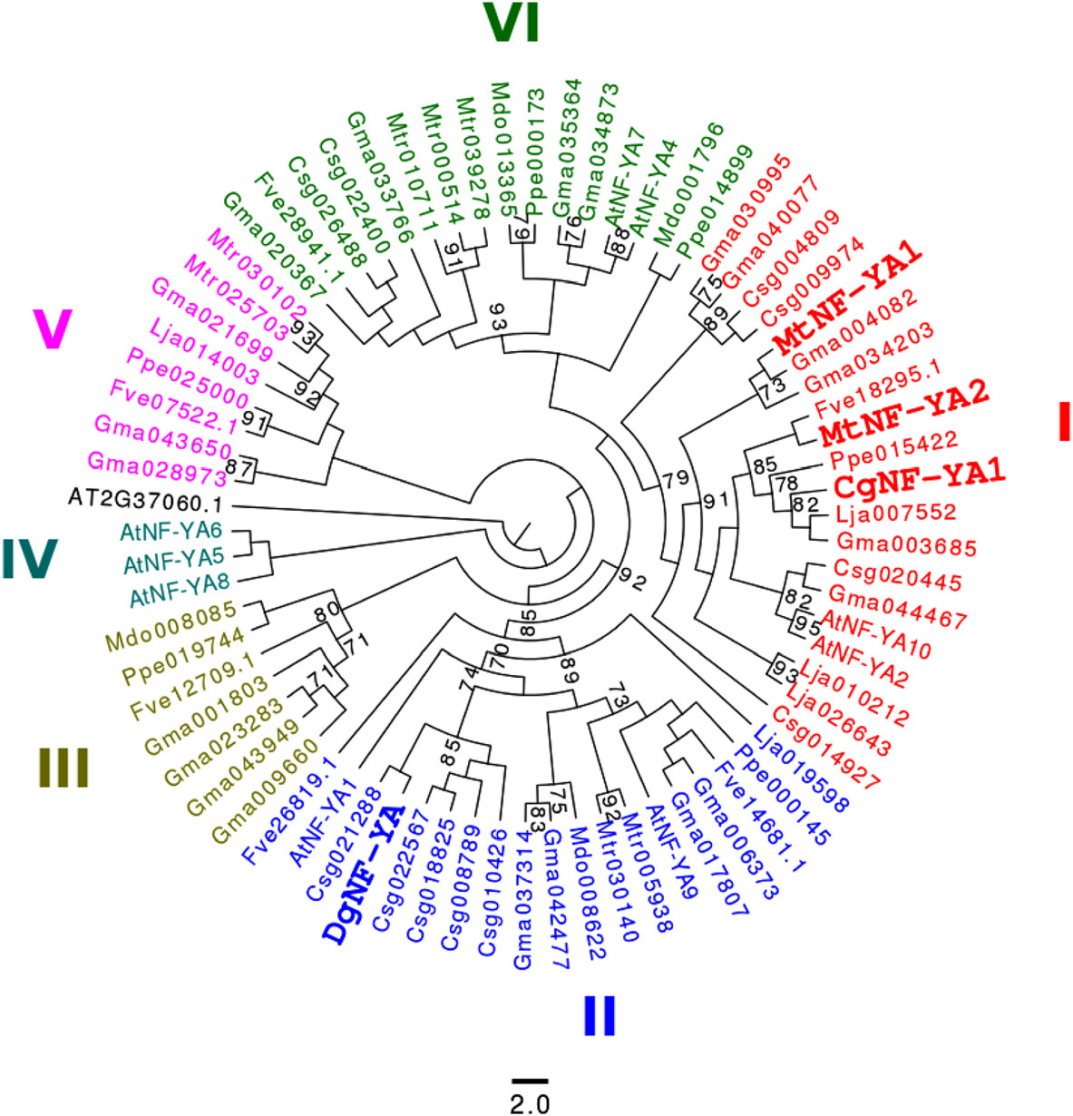
**Phylogenetic tree of the NF-YA protein family.** We include NF-YA proteins from *A. thaliana* (9 proteins), *M. truncatula* (9), *L. japonicus* (5) and *G. max* (21). Sequences of *C. sativus* (11), *P. persica* (6), *M. domestica* (4) and *F. vesca* (6) were retrieved using a BLAST-P search performed in phytozome and one sequence of *D. glomerata* (https://fido.nsc.liu.se/) [6]. In the present study, NF-YA tree comprises six groups: I to VI. *CgNF-YA1* belongs to Group I. *DgNF-YA* (comp3430_c0_seq7) is in Group II. The tree was rooted with *A. thaliana* sequence AT3G37650.1 (NF-YB). One hundred bootstrap replications were used to evaluate statistical support for branches. Branches with less than 70% bootstrap support were collapsed.

Plant genes belonging to the NF-YA family encode putative TFs that are variable in length [25]. Their DNA binding domain shares some similarity with the CCT domain of the nuclear flowering time regulator CONSTANS (CO) [26,27]. NF-YA proteins are characterized by two domains (protein binding and DNA binding domains) that are strongly conserved in all higher eukaryotes examined to date [25]. To clarify the phylogenetic situation of the NF-YA genes identified in the transcriptomes, we built a tree using the maximum likelihood method using only the NF-YA domains. Five of the originally collected sequences had incomplete DNA and protein domains in the *C. glauca* and *A. glutinosa* datasets and were excluded from further analysis. The NF-YA tree includes 6 major groups (Groups I–VI) also found in legumes (Figure 4, [25]). Interestingly, Group I containing *MtNF-YA1* and *MtNF-YA2* from *M. truncatula* includes *CgNF-YA1* from *C. glauca*. In the present study, *Dgcomp3430_c0_seq7* from *D.glomerata* is in Group II. Several genes belonging to Group II show a strong expression mainly in seeds [26,28].

**Figure 5:**
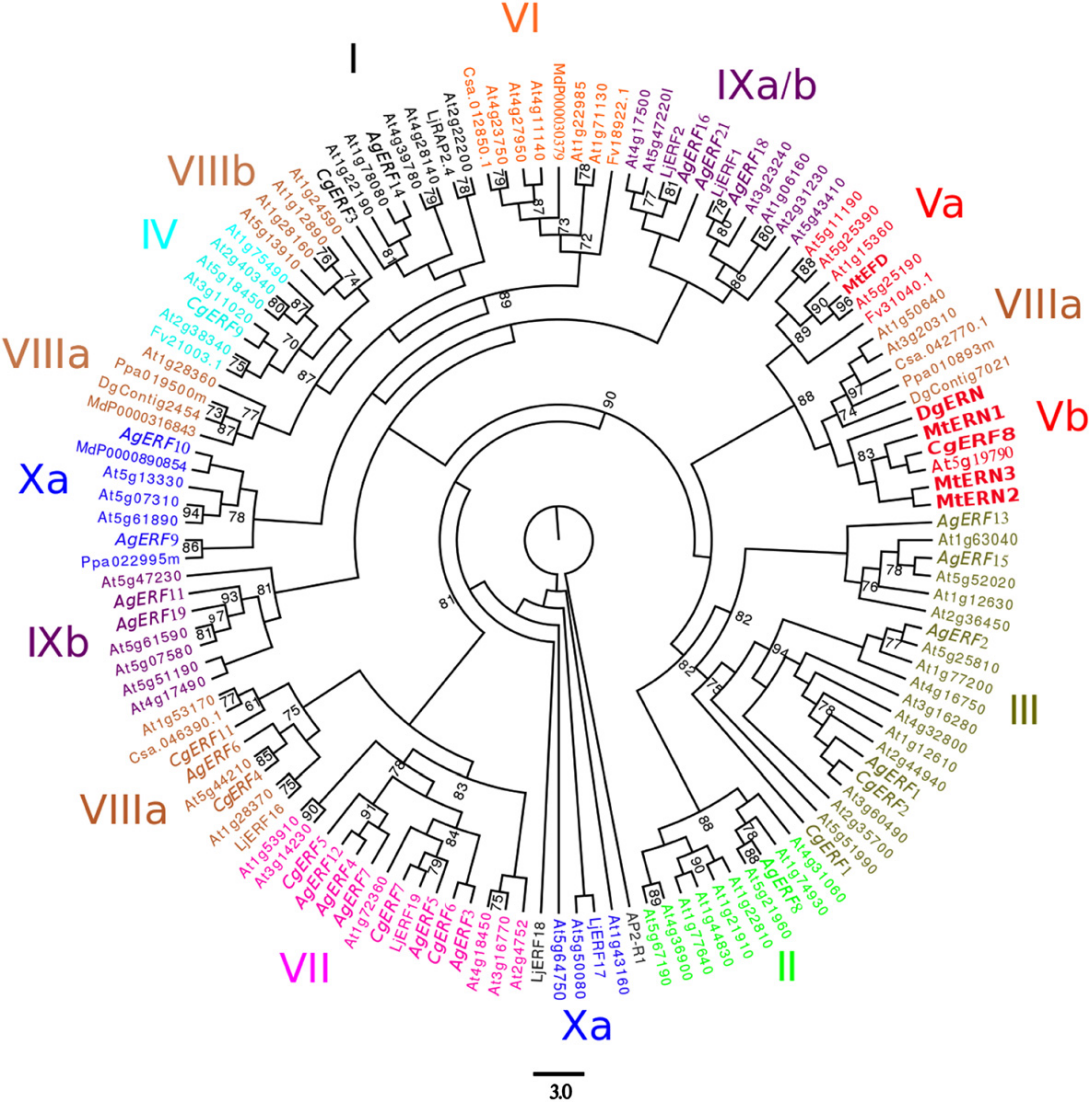
**Phylogenetic tree of the ethylene-response factor (ERF) protein family.** Three ERF proteins from *C. sativus*, *P. persica*, *M. domestica* and *F. vesca* each were retrieved using a BLAST-P search performed in phytozome and 2 sequences DgERN (comp6569_c0_seq1) and DgContig2454 (Contig2454) from *D. glomerata* (https://fido.nsc.liu.se/) [6]. We also include 74 ERF from *A. thaliana*, 4 from *M. truncatula* and 7 from *L. japonicus*. In italic, transcription factors *C.glauca* and *A.glutinosa*. The ERF family comprises ten groups referred as I to X. CgERF8 and DgERN are in Group V (red fat letters). They are closer to MtERN1 than MtERN2 and MtERN3. The tree was rooted to APETALA2 AP2-domain R1 (At4g36920). One hundred bootstrap replications were used to evaluate statistical support for branches. Branches with less than 70% bootstrap support were collapsed.

To study the phylogenetic relationships between the ERF genes in the actinorhizal species, multiple alignment analysis was performed using amino acid sequences in the AP2/ERF domain. Ten of the originally collected sequences had incomplete AP2 domains in the *C. glauca* and *A. glutinosa* datasets and were excluded from further analysis. The ERF tree distinguished the 10 major groups (Groups I–X) also found in rice, cotton, and Arabidopsis (Figure 5, [29,30]). At least one *C. glauca* and *A. glutinosa* ERF TF was found in the main groups except for Group VI. Group VII, which included five ERFs from *Arabidopsis*, was the most represented by the actinorhizal ERF transcription factor with three sequences in *C. glauca* and five sequences in *A. glutinosa*. However, several changes in the groups were observed (Figure 5). Group IX was divided into three subgroups [29]. In the present study, the phylogenetic tree showed that subgroup IXb was no longer related to subgroup IXa and IXc. Group IX contained five sequences from *A. glutinosa* and two sequences from the model legume *Lotus japonicus* including *LjERF1. LjERF1* functions as a positive regulator of nodulation [31]. *AgERF18* is the sequence most closely related to *LjERF1*. Group V was divided into two subgroups [29]. In the present study, the phylogenetic tree showed subgroups Va and Vb grouped with Group VIIIa. Interestingly, Group Vb which contained ERN1, ERN2 and ERN3 from *M. truncatula*, also includes two ERFs, CgERF8 and Dgcomp6569, from *C. glauca* and *D. glomerata,* respectively. The reliability of Group V clustering was supported by the presence of common motifs outside the AP2/ERF domain. CgERF8 and Dgcomp6569 as well as ERNs possess the CMV-3 or CMV-4 motifs, like in rice and *Arabidopsis* (data not shown) [29].

### C_2_H_2_ transcription factors in actinorhizal symbiosis

From the global analysis, the large number of its members and their regulation during actinorhizal symbioses make the C_2_H_2_ family particularly interesting. We therefore decided to study this family further, and in particular two of its members, *CgZF1* and *AgZF1*. Expression data showed that four transcription factors of this family were induced and seven less expressed in *C. glauca* nodules than in non-inoculated roots (Figure 1; Additional file 4). *CgZF1* was specifically induced during nodulation and no C_2_H_2_ were induced in *C. glauca* mycorrhized roots (Figures 1 and 2 and Additional files 3 and 4). Analysis of the *A. glutinosa* nodule transcriptome showed that one C_2_H_2_ was down-regulated and three were up-regulated in nodules (Figure 1; Additional file 8). Remarkably, like in the *C. glauca* TF gene expression dataset, a C_2_H_2_ TF, *AgZF1*, showed the strongest gene induction in *A. glutinosa* nodules as indicated by the red triangles in Figure 1. The C_2_H_2_ family is characterized by the presence of a “zinc finger” DNA binding domain containing one or two “QALGGH” conserved motif(s) [32]. CgZF1 contains two QALGGH motifs at positions 85–105 and 142–162. Furthermore, a putative EAR (ethylene-responsive element-binding associated amphiphilic repression) repressor domain with the signature (S)/(F)DLN(L)/(F)XP was identified at the C-terminal positions 176 and 180 of CgZF1. The “EAR domain” is known to be responsible for the repression of gene expression [33,34]. The presence of this domain in C_2_H_2_ C1-2i proteins suggests that they are transcriptional repressors of their target genes. Finally, a nuclear localization signal (NLS) involved in the translocation of protein to the nucleus was also identified at position 31–36 in its N-terminal end. Alignment of AgZF1, with the *C. glauca* CgZF1 revealed the position of DNA-binding including two “QALGGH” conserved motifs, an EAR domain and a NLS within the protein sequence and a high degree of identity (79%) with CgZF1 (Additional file 10).

**Figure 6:**
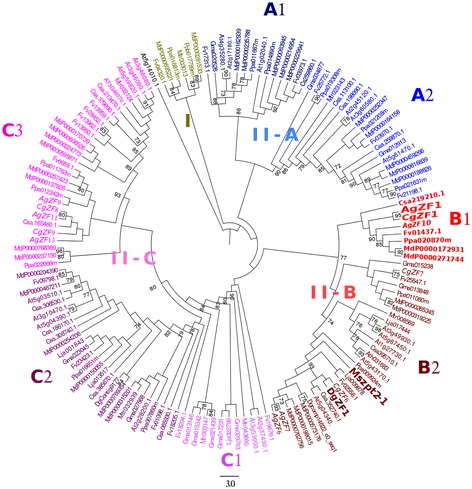
**Phylogenetic tree of the C**_**2**_ **H**_**2**_ **C1-2i protein family** ***.*** A maximum likelihood tree representing relationships among 24 C_2_H_2_ proteins from *A. thaliana*, 13 from *G.max*, 7 from *M. truncatula* and *A. glutinosa*, 5 from *L. japonicus* and *C. glauca,* 3 sequences DgZF1 (Contig7810), Dgcomp14622_c0_seq1 (comp14622_c0_seq1) and DgContig9177 (Contig9177) from *D. glomerata.* The tree was rooted with *A. thaliana* sequence AT5G14010.1 (C1-1i). In italic, transcription factors *C.glauca* and *A.glutinosa*. The C_2_H_2_ C1-2i family comprises two groups referred to as I and II. Group II is divided into three subgroups named A, B and C. The subgroups are also divided into several clusters. Cluster II-B1 contains sequences of actinorhizal plants and related sequences from Rosales and Cucurbitales include CgZF1 and AgZF1 (red fat writing). The sequence of *M. sativa* Mszpt2-1 was added to the dataset because it encodes a C_2_H_2_ C1-2i type transcription factor. The nodes represent bootstrap values (≥ 70%) from 100 replications.

To further characterize the relationship between CgZF1 and AgZF1, a phylogenetic tree based on the alignment of the complete protein sequences was generated (Figure 6). The sequence of *M. sativa* Mszpt2-1 was added to the dataset because it encodes a C_2_H_2_ TF and plays a role in legume symbiosis [35]. The phylogenetic tree distinguished two major groups, Groups I and II. Group I did not contain *C. glauca*, *A. glutinosa* and *A. thaliana* C_2_H_2_ TF. Group II was divided into three subgroups named A, B and C. Subgroups II-B and II-C contained seven and five C_2_H_2_ TFs from actinorhizal plants, respectively. Interestingly, one cluster in subgroup II-B containing *CgZF1* and *AgZF1* was only made up of sequences from Rosales, Fagales, and Cucurbitales and did not contain any legume sequence. Our analysis also confirmed that CgZF1 and AgZF1 protein sequences are very close and are most probably orthologs (79% similarity, data not shown; bootstrap value, 95%, Figure 6). This suggests that ZF1-like transcription factors evolved after the divergence between Fabales and the Rosales/Cucurbitales/Fagales clade and the common ancestor of actinorhizal Fagales recruited these genes for functions related to nodulation.

## Discussion

The search for putative transcription factors is an important first step in characterizing the signaling pathways that control the establishment and functioning of actinorhizal symbioses. This analysis enabled us to identify 202 and 195 transcription factors in *C. glauca* and *A. glutinosa,* respectively. The use of transcriptome data generated in *C. glauca* showed that half the putative transcription factors are regulated in nodules and mycorrhizae (Additional file 11). In *A. glutinosa*, 26% are significantly regulated in nodules. In addition, the transcriptome data pointed to co-regulation of *CgZF1* and *AgZF1,* which belong to the C_2_H_2_ family.

*In silico* studies have already been conducted using several plant models to optimize automated annotation of transcription factors and to obtain a plant TF atlas. For example, 2,023 transcription factors have been identified in *A. thaliana*, 1,613 in *M. truncatula*, 1,275 in *L. japonicus* and 3,557 in *G. max* [36]. These transcription factors are characterized by their DNA binding domains, which are specific to each family. This led to the identification of 58 families in *A. thaliana*, 56 in *M. truncatula* and *L. japonicus* and 57 in *G. max* [36]. The putative transcription factors are distributed in 40 families in *C. glauca* and 35 in *A. glutinosa*. The difference between these and other plant models can be explained by the fact that *C. glauca* and *A. glutinosa* genomic data come from 14,327 unigenes for *A. glutinosa* and 14,868 unigenes for *C. glauca*. As part of the effort to better understand the biology of actinorhizal symbiosis, large-scale cDNA sequencing using next generation sequencing technologies will provide a means to identify new TF genes and to produce an actinorhizal gene expression atlas.

Among the transcription factors identified, *CgNIN* (*Casuarina glauca nodule inception*) showed high similarity with *MtNIN* (*Medicago truncatula nodule inception*) and *LjNIN* (*Lotus japonicus nodule inception*). *CgNIN* was strongly induced in nodules (fold change = 27) and not significantly induced in mycorrhized roots (Figures 1 and 2). We also identified another gene called *CgERF8* coding for an ERF transcription factor. This gene was induced in both *C. glauca* nodules and mycorrhizae. In *M. truncatula*, a gene encoding a transcription factor belonging to the ERF family named *MtERN2* [37] presents the same expression profile as *CgERF8*. These two genes share 55% similarity and conservation of specific domains (data not shown). In addition, we identified *CgNF-YA1*, a close homologue of *MtNF-YA1* and *MtNF-YA2*, two TFs involved in the activation of *MtERN1* [20]. *CgNF-YA1* is induced in actinorhizal nodules (fold change = 3,296, see Figure 1). No sequence of *C. glauca* belongs to the NSP1 and NSP2 groups. In *A. glutinosa*, AgGRAS7 belongs to the NSP1 group and is closely related to DgNSP1 (bootstrap value = 71%). Consequently, we suggest that *CgNIN, AgNSP1*, *CgNF-YA1* and *CgERF8* could be functional homologs of *NIN, NSP1*, *NF-YA* and *ERN1* in legumes. Functional complementation of legume mutants by the potential actinorhizal orthologous genes could be used to test this hypothesis.

Analysis of the expression of the putative transcription factors was performed using transcriptome data to investigate their expression during the *C. glauca-/A. glutinosa-Frankia* interactions. This investigation revealed 25 induced TFs in *C. glauca* or *A. glutinosa* nodules. Recently, 192 genes encoding putative transcription factors strongly induced in nodules were identified in *M. truncatula* [38]. These TFs were classified in eight functional groups of genes differentially regulated in *M. truncatula* nodules in seven experimental conditions after inoculation with the wild type and three *Nod-* mutant strains of *Sinorhizobium meliloti*. Transcription factors belonging mainly to the C_2_H_2_, MYB, WRKY and ERF families appeared to be nodule-specific, whereas members of the ERF and WRKY families were over-represented in the TF dataset [38]. Similarly, transcriptome analysis of *L. japonicus* led to the identification of 20 TF that expression was up-regulated during nodulation showing that members of the ERF family are the most abundant TF [31]. Remarkably, members of the ERF and WRKY families of TF were also abundant in our study, and expression analysis showed that nine of them were induced in actinorhizal nodules. ERF and WRKY TFs are often involved in defense responses to abiotic and biotic stresses [39-41]. As hypothesized for legume and rhizobium symbioses, ERF and WRKY TFs could be involved in the regulation of downstream defense responses to inhibit excess *Frankia* infection under nitrogen-sufficient conditions.

Three genes named *CgZF1*, *CgHD-ZIP2* and *CgG2-like5* belonging to the C_2_H_2_, HD-ZIP and G2-like families, respectively, are specific to the *C. glauca-Frankia* interaction. A TF named *AgZF1* exhibited the same strong induction in *A. glutinosa* nodules as *CgZF1*. In addition, these two genes were also seen to have the same protein structure and phylogenetic analysis indicate they are most likely orthologous. In legumes, two C_2_H_2_ TFs are involved in nodule functioning. In *M. truncatula*, MtRSD represses transcription of the secretory pathway gene, *VAMP721a,* required for symbiosome formation, suggesting that MtRSD is directly involved in symbiosome and bacteroid differentiation [21]. MtRSD is a C_2_H_2_ TF but is phylogenetically distant from CgZF1 and AgZF1 (data not shown). In *M. sativa*, a gene called *Mszpt2-1* is involved in the differentiation of nitrogen-fixing cells [35]. Mszpt2-1 possesses two “QALGGH” motifs and an EAR motif as well as CgZF1 and AgZF1. Mszpt2-1, CgZF1 and AgZF1 belong to the same C_2_H_2_ Group II (Figure 6). Functional complementation studies will determine whether *CgZF1* and *AgZF1* are functionally equivalent to putative orthologous genes identified in legumes. Recent studies showed that repressors containing the EAR motif play a role in defense responses to plant biotic and abiotic stress [42]. CgZF1 and AgZF1 could repress the expression of defense genes of actinorhizal plant in response to signals from *Frankia* to allow colonization of new cells in nodules. Finally, *CgZF1* and *AgZF1* could be used as functional markers to investigate the regulation of actinorhizal symbiosis interaction.

## Conclusions

The *in silico* identification and transcriptome analysis of TFs in *C. glauca* and in *A. glutinosa* that we carried out represents a crucial step forward in the elucidation of the molecular events underlying actinorhizal and mycorrhizal symbioses. Coupled with a phylogenetic study, these analyses enabled identification of a nodulation specific gene in *C. glauca* named *CgZF1* and its ortholog in *A. glutinosa* named *AgZF1*. These two new genes are most probably negative regulators which may play a crucial role in actinorhizal symbiosis. The development of efficient techniques to transform *C. glauca* [43] now allows us to perform functional studies of *CgZF1*.

### Availability of supporting data

EST sequences reported in this paper have been deposited in the GenBank (accession nos. CO036851–CO0388878) and EMBL (accession nos. FQ312199–FQ377516) databases. The normalized and raw microarray data values have been deposited in the Gene Expression Omnibus database (www.ncbi.nlm.nih.gov/geo; accession nos. GPL10929 and GSE24153 for *C. glauca* and *A. glutinosa* respectively).

In addition, all nucleic acid sequences used in this study have been included in Additional files 1 and 2. Proteins sequences used to produce phylogenetic data sets have been added in Additional file 12.

## Methods

### Database search

Fifty-eight predicted DNA-binding domain protein sequences from *Arabidopsis* were used as query sequences for tBLASTn searches against the predicted *C. glauca* and *A. glutinosa* proteins in a previously described database (http://www.ncbi.nlm.nih.gov/nucest/; Accession numbers CO036851 to CO0388878 and FQ312199 to FQ377516) [5]*. Arabidopsis* DNA-binding domain sequences were retrieved from a multi-alignment available in DATF (Database Arabidopsis Transcription Factors: http://planttfdb.cbi.edu.cn/ version 2.0). The TAIR database (http://www.arabidopsis.org/Blast/) was used to confirm the affiliation of each putative transcription factor to one of the 58 families. tBLASTx were carried out to check trans-species sequence homologies between genes with an e-value cut-off of 1e^−10^ and genes were annotated according to the TAIR database.

### Expression data of putative TF in actinorhizal nodules and mycorrhizal roots

The putative TF expressed in *C. glauca* (Accession number GPL10929) and in *A. glutinosa* (Accession number GSE24153) were retrieved from microarray data [5,7]. A transcription factor was considered differentially expressed if its fold change was greater than or equal to 2 and its p-value lower than or equal to 0.01.

For quantitative RT-PCR analyses, total RNA was purified from roots and nodules by ultracentrifugation [44] for *C. glauca* and using the RNeasy plant mini kit (Qiagen, Courtaboeuf, France, see [45]) for *A. glutinosa*. RNA was quantified using a Nanodrop (Thermo Fisher Scientific, Courtaboeuf, France) and analyzed using a Bioanalyzer 2100 according to the manufacturer’s instructions (Agilent, Santa Clara, CA, USA). For *C. glauca* analyses, reverse transcription were performed on 0.5-1 μg of RNA using the SuperScript® reverse transcriptase III kit (Invitrogen Life Science, Carlsbad, CA, USA). For *A. glutinosa* analyses, reverse transcription was performed with 9 μg of RNA using Transcriptor Revers transcriptase and oligo(dT)_15_ primer (Roche, Mannheim, Germany). Reverse transcription (RT) and real time quantitative PCR (qRT-PCR) were performed with the same three biological replicates of nodules and non-inoculated roots. Primers were designed using Beacon designer software (Premier Biosoft International, Palo Alto, CA, USA). Quantitative RT-PCR analyses were conducted as previously described [5] using primers listed in supporting information (Additional file 13). For *C. glauca*, amplifications were performed using a Stratagene Mx3005P thermal cycler, (Agilent, Palo Alto, CA, USA) with the Brilliant II SYBR Green QPCR Master Mix (Agilent, Palo Alto,CA, USA) programmed for a pre-denaturation step of 5 min at 95°C followed by 40 cycles of 10 s at 95°C and 30 s at 60°C and 72°C for 15 s . For *A. glutinosa*, amplification was run on a LightCycler 480 (Roche) using LightCycler 480 SYBR Green I Master (Roche) under the following conditions: 95°C for 5 min; 45 cycles of 95°C for 20 s, 60°C for 20 s and 72°C for 15 s. The *C. glauca ubiquitin* gene (*CgUbi)* and the *A. glutinosa* gene (*AgUbi*) were used as controls as reported in [44] and, respectively [5].

### Semi-quantitative PCR analysis

*cDNA* from *C. glauca* uninfected roots, nodules, non-mycorrhizal roots and mycorrhizae were obtained by reverse transcription RNA as described in [5]. *CgZF1* was amplified by PCR using specific sense and antisense primers *CgZF1* (Additional file 13). *CgUBI* was used as control. Amplification was performed using a *GeneAmp*® *PCR* System *2400* (Perkin Elmer) programmed for a pre-denaturation step of 2 min at 94°C followed by 32 cycles of 30 s at 94°C and 30 s at 62°C and 72°C for 45 s.

### Phylogenetic relationship between transcription factors

Phylogenetic analysis was performed using the maximum likelihood method on protein sequences from the four TF families: C_2_H_2_, ERF, GRAS and NF-YA, identified in *C. glauca* and *A. glutinosa*. We used the default algorithms and settings implemented in www.phylogeny.fr. Briefly, a protein-based alignment of full-length sequences or conserved domains was generated using MUSCLE [46], this alignment was then curated using the Gblocks [47] to remove poorly aligned positions and gaps and a phylogenetic tree was calculated using PhyML [48]. Trees were edited with FigTree (“http://tree.bio.ed.ac.uk/software/figtree/”). We used AT5G14010.1, AP2-R1, AT4G36710.1 and AT2G37060.1 to root the C_2_H_2_, ERF, GRAS and NF-YA trees respectively. Trees were constructed based on sequence alignments of DNA binding domains for ERF and NF-YA and complete sequences for C_2_H_2_ and GRAS. The DNA binding domains or protein sequences of these four TF families from *A. thaliana* (http://planttfdb.cbi.edu.cn/index.php?sp=At)*, M. truncatula* (http://planttfdb_v1.cbi.pku.edu.cn:9010/web/index.php?sp=mt), *L. japonicus* (http://planttfdb_v1.cbi.pku.edu.cn:9010/web/index.php?sp=lj), *G. max* (http://planttfdb.cbi.edu.cn/index.php?sp=Gma), *Cucumis sativus*, *Prunus persica*, *Malus domestica* and *Fragaria vesca* (http://www.phytozome.net/search.php?show=blast&method=Node_rosales-cucurbitales) were added to the dataset. The bootstrap values represent a percentage of 100 repetitions.

## Additional files

Additional file 1:
**Potential transcription factors in** ***C. glauca*** **.**
Additional file 2:
**Potential transcription factors of** ***A. glutinosa.***
Additional file 3:
**Putative transcription factors up and down-regulated in** ***C. glauca*** **nodules.**
Additional file 4:
**Distribution of transcription factors families regulated in** ***C. glauca*** **nodules and Mycorrhizae.**
Additional file 5:
**Validation of few transcription factors up regulated in** ***C. glauca*** **and** ***A. glutinosa*** **nodules by real time quantitative PCR.**
Additional file 6:
**Validation of transcriptome data by RT-PCR. NR = non-inoculated root, NOD = Nodule, NMR = Non-mycorrhizal root and MR = mycorrhizae.** *CgZF1* = *C. glauca Zinc Finger 1, CgUBI* = *C. glauca Ubiquitin* (Control). Expression data from microarray of CgZF1 were confirmed by semi-quantitative RT-PCR. The expression of *CgUBI* is constitutive and standardizes the expression of *CgZF1.*
Additional file 7:
**Putative transcription factors up and down-regulated in** ***A. glutinosa*** **nodules.**
Additional file 8:
**Distribution of transcription factors families regulated in** ***A. glutinosa*** **nodules.**
Additional file 9:
**Putative transcription factors up and down-regulated in** ***C. glauca*** **mycorrhizae.**
Additional file 10:
**Multiple sequence alignment of CgZF1 and ZF1-like proteins from** ***Alnus glutinosa, Cucumis sativus, Fragaria vesca, Prunus persica*** **and** ***Malus domestica*** **.**
Additional file 11:
**Percentage of transcription factors regulated in** ***C. glauca*** **nodule and mycorrhizae.**
Additional file 12:
**Protein sequences of the GRAS, NF-YA, ERF and C**_**2**_ **H**_**2**_ **C1-2i TF used to generate the phylogenic trees.**
Additional file 13:
**Primers list for semi or quantitative PCR.**

## Abbreviations

EST: Expressed sequence tag
PCR: Polymerase chain reaction
qRT-PCR: Quantitative real time-polymerase chain reaction
TAIR: The Arabidopsis Information resource
BLAST: Basic local alignment search tool
NCBI: National Center for Biotechnology Information
DATF: The Database of Arabidopsis Transcription factors
CDS: Coding sequence
