# Supplementary material for: Identification of potential transcriptional regulators of actinorhizal symbioses in Casuarina glauca and Alnus glutinosa
Source: BMC Plant Biol. 2014 Dec 10;14:342. doi: 10.1186/s12870-014-0342-z (PMC4264327; doi:10.1186/s12870-014-0342-z)
Supplement: Additional file 6: — Validation of transcriptome data by RT-PCR. NR = non-inoculated root, NOD = Nodule, NMR = Non-mycorrhizal root and MR = mycorrhizae. CgZF1 = C. glauca Zinc Finger 1, CgUBI = C. glauca Ubiquitin (Control). Expression data from microarray of CgZF1 were confirmed by semi-quantitative RT-PCR. The expression of CgUBI is constitutive and standardizes the expression of CgZF1. [file 12870_2014_342_MOESM6_ESM.ppt]

## Slide 1
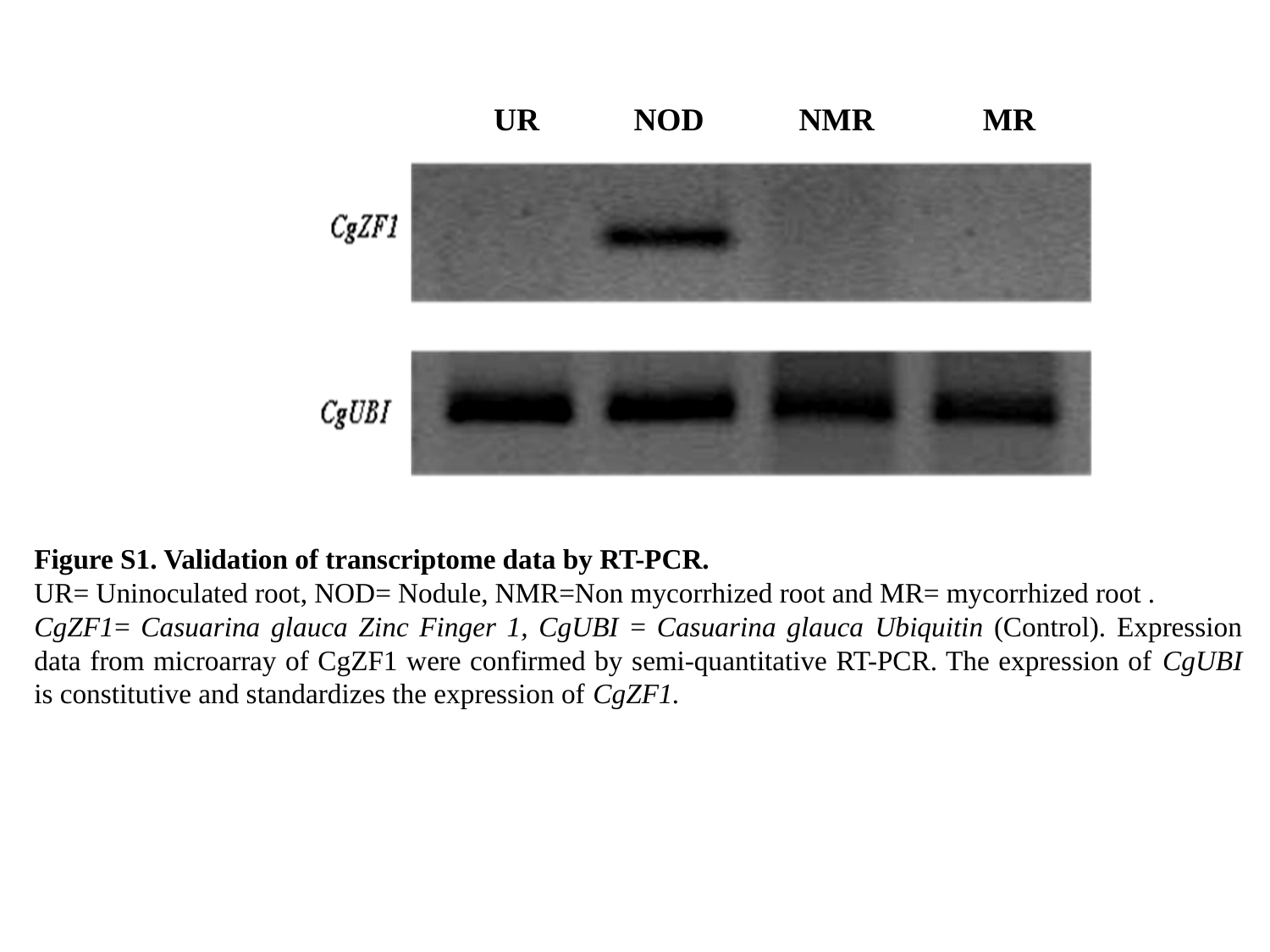

UR
NOD
NMR
MR
Figure S1. Validation of transcriptome data by RT-PCR.
UR= Uninoculated root, NOD= Nodule, NMR=Non mycorrhized root and MR= mycorrhized root .
CgZF1= Casuarina glauca Zinc Finger 1, CgUBI = Casuarina glauca Ubiquitin (Control). Expression data from microarray of CgZF1 were confirmed by semi-quantitative RT-PCR. The expression of CgUBI is constitutive and standardizes the expression of CgZF1.
