# Supplementary material for: Identification of potential transcriptional regulators of actinorhizal symbioses in Casuarina glauca and Alnus glutinosa
Source: BMC Plant Biol. 2014 Dec 10;14:342. doi: 10.1186/s12870-014-0342-z (PMC4264327; doi:10.1186/s12870-014-0342-z)
Supplement: Additional file 10: — Multiple sequence alignment of CgZF1 and ZF1-like proteins from Alnus glutinosa, Cucumis sativus, Fragaria vesca, Prunus persica and Malus domestica . [file 12870_2014_342_MOESM10_ESM.doc]

**NLS Motif**

Csa.219210.1 ----------MALQALNSPSSTFPL-ADPSLDHHHHDSWIKPSKPR----SKRPRFDSD-

CgZF1 ----------MALEALSSPISAAPLLHNDRAEDRYLGPWVKSKRSK------RPRLHNPA

AgZF1 ----------MALEALSSPISAAPLSHNDSAEDHYVELWAKSKRSK------RPRLDNPP

Fv01437.1 MDMEHQEITNPDIT--------------ESNNNNGGGLLIKLKMAKAEPFSEQGNRKPET

AgZF10 -----GKIKFVDLEAL-----------------RDGG--VQSKKQT--------------

Ppa020870m MDVEDQETRFRP-RAGDQEQQPNKK-TNQETESNNGGLFAKLKFPKPEPLDQERKYPAP-

MdP0000271744 MDIEHQETRFPPLTASDQERKPAKILSREEMESNNEGMFVKLKVAKPEPLGQNPQNFSAA

MdP0000172931 MDIQHKETRFPPLAARYQEPTPAKKPSREEMENNNGGLFLELKVAKPEPLDQNPQNSSAX

Csa.219210.1 --DEYLAFCLLML---ARGRISHSDHHHH-------------------------------

CgZF1 AEEEYLALCLLML---GQG-----------------------------------------

AgZF1 TEEEYLALCLLMLGGQGSG-----------------------------------------

Fv01437.1 LAPRFTKICHICQKGFESGKALGGHMRMHVQESKGL--------IKSSSKSPKPNSAT--

AgZF10 ------------------------------------------------------------

Ppa020870m --PEFTRICEVCNKGFSSGKALGGHMRMHVQANRELF-QARKNKIKKPTKILKPNNSDVS

MdP0000271744 PLPEFTRFCEVCFKGFSSGKALGGHKRMHVQANRELFNQSRKNTISSKPNNSKPSNSNVG

MdP0000172931 PRPQFTRFCEVCGKGFSSGKALGGHMRIHIQADRELFNQSXKNMISSKPKISKPNNSDVG

Csa.219210.1 ------------HATTNDSYSPSNSS-------------------------PPPPPLL--

CgZF1 ----------GATPTTNTENQPP----------------------------LTPFSQ---

AgZF1 ----------GATTTTNTKNQPC----------------------------PTMPSQ---

Fv01437.1 --------ELFSTSATDSGGEHVCIECGKIFPSKKSLYGHMRSHPDRPYRGIEPPTQFSK

AgZF10 ---------------------------------------------------IPVTPN---

Ppa020870m SSNGGGGFGVEGNSNSNSSMKPDCCVCGKNFPSMKSLFGHMRSHPEREWRGIQPPPTIAK

MdP0000271744 SSNAGGVLGIGGNSSTISNMKPVCCVCGKNFPSMKSLFGHMRSHPEREWRGIQPPPT-AK

MdP0000172931 SSNAGGVVGIGGNSNSISDXKPVCCVCGKNFPSMKSLFGHMRSHPEREWRGIQPPPA-AK

Csa.219210.1 ------------------------------------------------------------

CgZF1 ------------------------------------------------------------

AgZF1 ------------------------------------------------------------

Fv01437.1 NSSTSTLSDALP-QKPNLAEDQQIDSAGTARVWFGPNPKAVRDLVKVVPSWSKTAKRGRK

AgZF10 ------------------------------------------------------------

Ppa020870m NSSSSTLSDAVP-QNNNKADDHQIDSAAT----PVGSNNSAPDLSKTLPGWSLTARRGRK

MdP0000271744 NSSSSTVSDAVPDQSNRKAQEDQIESDET----FFRSKSSVLNLSKTLPKWSQTARRGRK

MdP0000172931 NSSSSTLSDAVPDQSEKKDQDGQIDSDEM----FFASKSSALDLSKTLPKWSQTARRGRK

Csa.219210.1 ------------------------------------------------------------

CgZF1 ------------------------------------------------------------

AgZF1 ------------------------------------------------------------

Fv01437.1 --GTSSLDSETDSESDDDDCADD---EDEELDMSEAVHDLMMLAQANP-------KGAE-

AgZF10 ------------------------------------------------------------

Ppa020870m SIGSSG----------------------NSESMEEAVHDLLMLAQSNPCFYGLSDKGKGA

MdP0000271744 S-GSTGFESNSDQSVSGLDSRSPRTVEEDEEQMEEAVHDLLMLAQANP----LEGGGADE

MdP0000172931 S-GSTGLESNSDQSVSGLDSRSPRTVEEDEEQMKEAVHDLLLLAQANP----LEGGGADD

Csa.219210.1 ------------------------------------------------------------

CgZF1 ------------------------------------------------------------

AgZF1 ------------------------------------------------------------

Fv01437.1 ---EATNSNFMTKNNQDHD---VKDIRVIDYL-GVAKKRKKLEIEEDGDLEKP-------

AgZF10 ------------------------------------------------------------

Ppa020870m EMCEATNSNFFITNNSNQADYIKDKNQVIDYE-GTSKKRKGTEMCFPSKNMKSEKKWFDD

MdP0000271744 TMYEVTNSNFLITTNQDREGGLPYHGHTAPSTVGSTNKRKGA----SSEVKRSEKKWYDA

MdP0000172931 TMCEATNSNFLITANQDGEXGLPCHGYTATATVGSTNKRKGS----PSEVERSEKKWYDE

Csa.219210.1 ------------------------------------------------------------

CgZF1 ------------------------------------------------------------

AgZF1 ------------------------------------------------------------

Fv01437.1 ---------------------RNQLELGLDLKY------DSEEELSDSENSESIVLANMM

AgZF10 ------------------------------------------------------------

Ppa020870m YDGMKGKGVLGIGFGPEKPSVKNFSKLGPMEDN---KNNNIEEELSDSQNSESIVLAS-M

MdP0000271744 YGG---------GFGQEKSTVKNFLKLGLMEEENIGNYNNMEDELSDSQNSESIAVV---

MdP0000172931 YGG---------GFGQEKSTVKNFLKLGLMEEENIGNHNNMEDELSDSQNSESIAVM---

**First zinc finger domain**

Csa.219210.1 --------------------------------KLTYNCNVCNKSFSSYQALGGHKASHRK

CgZF1 --------------------------------TLPYKCTLCHKAFPSYQALGGHKASHRR

AgZF1 --------------------------------TLPYKCTLCHKAFPSYQALGGHKASHRK

Fv01437.1 KRKKRRKMKLEDLDG-----AVVVDHQHQSHHHKVYTCSLCNKSFQSHQALGGHMSSHNK

AgZF10 ----------------------------------RYICCVCNKSFPSHQALGGHKSSHNK

Ppa020870m KRRKRRKMKLIDLEGVVGEIIGAQGHHQSLHQKLRYKCSLCGKSFPSHQALGGHMSSHNK

MdP0000271744 -RRKRRKMKLVDLEG-----------------RLRYKCNLCAKCFPSHQALGGHMSSHNK

MdP0000172931 -RRNRRKMKLVDLEG-----------------RLRYKCNLCVKCFPSHQALGGHMSSHNK

Csa.219210.1 SD-----------AGDNNVSPVVSSTL--------SNSTL--------------------

CgZF1 PI-----------GPEEQSFATTTTST---IMTNSKTSSL--------------------

AgZF1 PV-----------GLEDQCSTTTTTTT---AITTNSSNAL--------------------

Fv01437.1 LKNNNNSNNIQYSAEHDHHHQSGSEDTNN-ANANLTNTATQID-----------------

AgZF10 LK--------YIQTTVNESVSADDSAAED-CGGHYAEPTTQVD-----------------

Ppa020870m LKNNNNNIIVNHSSLDDQSASADVSAAEGGHNLDEAQTTMALDQDHHVAGAGSGSGAGGL

MdP0000271744 LK----GITVAHSSMEDQSAXADDPAAEDRVHGDEAERVA--------------------

MdP0000172931 LK----GIAVAHPSMEDQSAPADVSXGEDRVHDDEAERVA--------------------

**Second zinc finger domain**

Csa.219210.1 GGGVKTHQCSICFKCFPTGQALGGHKRRHYDGGSGNNN----------------------

CgZF1 NPSCKTHKCGICHRTFQSGQALGGHKRCHYDG----------------------------

AgZF1 NPNGKTHKCAICHKTFQSGQALGGHKRCHYDG----------------------------

Fv01437.1 HDQAESHQCRICNRTFLTGQALGGHMRSHWNGQNDQAQ----------------------

AgZF10 EATAGTHQCKICDKTFPTGQALGGHKRCHWTAGPVELQSSTQATSPGEAKAQSSTQETSP

Ppa020870m GAHQAHHQCKICDKIFPTGQALGGHKRCHWTGPTEQLL----------------------

MdP0000271744 LDHLQHHQCKICNKTFPTGQALGGHKRCHWTGPTEQQQQQVVVVGPSEQLXKQVVVVGPS

MdP0000172931 LDHQQHHQCKICNKTFPTGQALGGHKRSHWNGPTEQHQQQVV--------------EGPP

**EAR Motif**

Csa.219210.1 TNSTAATAGSDGNGSTLTQTHHRNFDLNIPALPELWPGFTAGNRRKKSQSQSQEYSTD--

CgZF1 --------------VITLSHSHRSFDLNFPALPEF-------------------------

AgZF1 --------------VITVSHSHRDFDLNFPALPEF-------------------------

Fv01437.1 ----------------SSQTGRKGLDIDLNELPPMEYEQGID------YSGAGYATSS--

AgZF10 EEAKAQSSQVTSPG-EASQTGRRILDFDLNELPAIELEEA---------GIAESVICM--

Ppa020870m ----PQSSQAPSPGEASQNAGRKVLNFDLNELPATEFEEGTD-----QYGAAGYATSS--

MdP0000271744 EQLQXQLQAHSSQTIVGGRGGGKVLNFDLNELPPMEEDNQEGAATKPQQNPTPTHKSSRP

MdP0000172931 EQLQQQLQAQSSQNIAGVGGGRRVLDFDLNELPPMEEDDQQGEGVGYQYGGAAAAGCAPS

Csa.219210.1 --------QEVESPHPLKKPKLLLPME----------

CgZF1 -------------------------------------

AgZF1 -------------------------------------

Fv01437.1 --------YNSINLS----------------------

AgZF10 --------YD---------------------------

Ppa020870m --------HNSVT------------------------

MdP0000271744 KPVFSLHFYNLEPGNPSFQLVVLHPVQDEARKPSEEP

MdP0000172931 S-------YNSVSN-----------------------

**Additional file 11: Multiple sequence alignment of CgZF1 and ZF1-like proteins from *Alnus glutinosa, Cucumis sativus, Fragaria vesca, Prunus persica* and *Malus domestica*.**
