# Supplementary material for: Identification of potential transcriptional regulators of actinorhizal symbioses in Casuarina glauca and Alnus glutinosa
Source: BMC Plant Biol. 2014 Dec 10;14:342. doi: 10.1186/s12870-014-0342-z (PMC4264327; doi:10.1186/s12870-014-0342-z)
Supplement: Additional file 11: — Percentage of transcription factors regulated in C. glauca nodule and mycorrhizae. [file 12870_2014_342_MOESM11_ESM.ppt]

## Slide 1
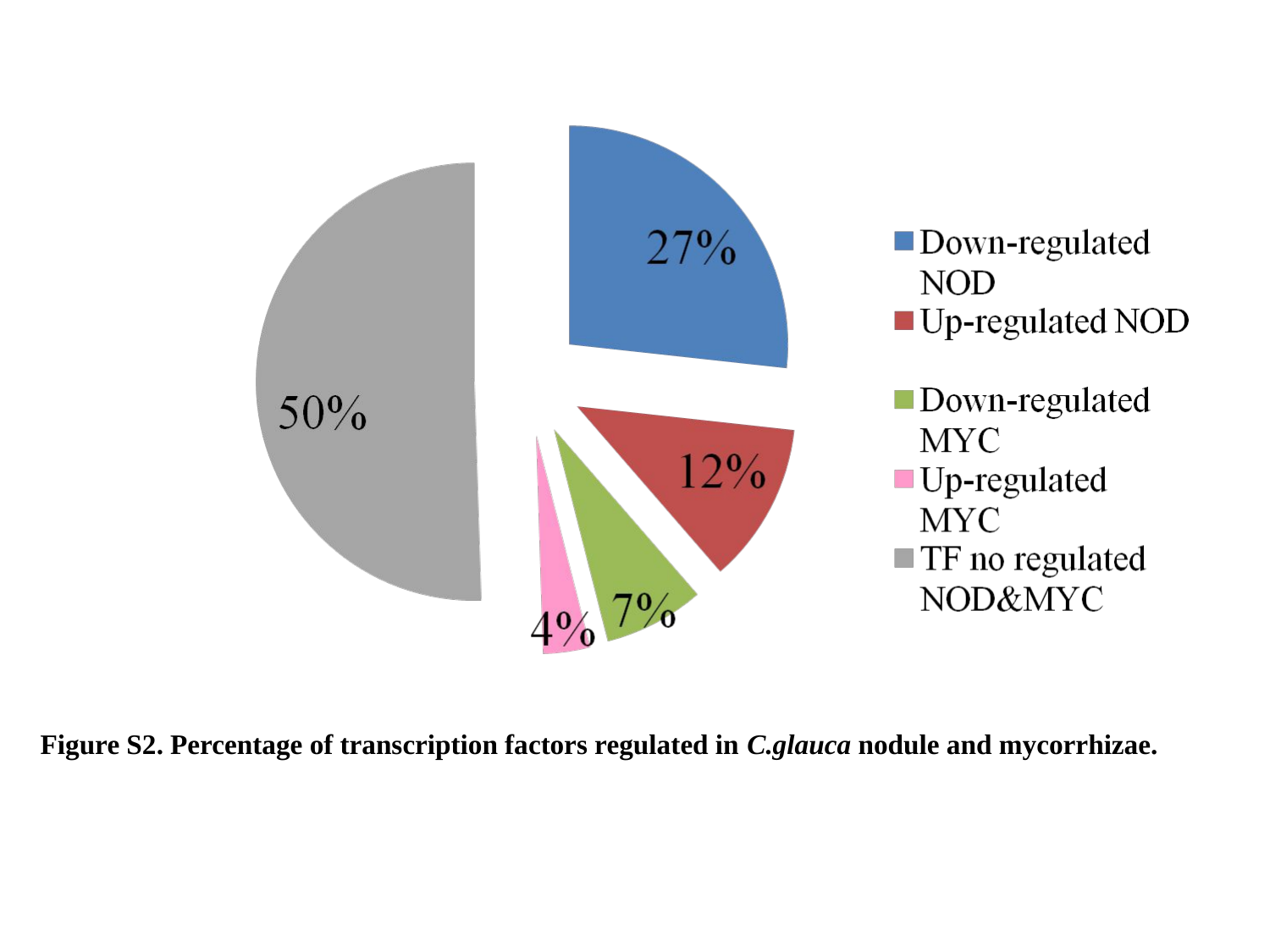

Figure S2. Percentage of transcription factors regulated in C.glauca nodule and mycorrhizae.
