# Supplementary material for: Identification of potential transcriptional regulators of actinorhizal symbioses in Casuarina glauca and Alnus glutinosa
Source: BMC Plant Biol. 2014 Dec 10;14:342. doi: 10.1186/s12870-014-0342-z (PMC4264327; doi:10.1186/s12870-014-0342-z)
Supplement: Supplementary file 12 [file 12870_2014_342_MOESM12_ESM.doc]

## Sheet: Feuil1

Additional file12.PrimerslistforsemiorquantitativePCR
F=Forward
R=Reverse
Sequence name	Accession number	Gene	Oligo sequence (5' to 3')
CGCL2898Contig1	CO038838.1,FQ372784	CgbHLH4	F-CAGTGACCAAGCCTACCCATTTC                                    R-CAAAGTCTTCATCCTCCACCATCG
CGCL2533Contig1	FQ313294,FQ318868	CgMyb-related3	F-CCGTCTTCTTCATTGTCCTCCTC'                         R-GAACTCCCTTCTTCCTCTCTTGG
CG-R02f_046_A16	FQ327959	CgWRKY15	F-CTGAACTTGTCTCACTTCGTCTCG                      R-TGCTTAGGCTCTTCCAAACTACCC
CGCL1984Contig1	FQ322708,FQ317687	CgbZIP8	F-GCGAGGAGGTCCCGAATCG'                                  R-GTGGTGTGGTTTATGCTTGTCAGG
CG-N02f_013_O07	FQ365058	CgbHLH8	F-CCCAAGTCCATCTCCTCCAAAC                               R-GATAAGCCATCCTCCTCAGACC
CG-R02f_040_B14	FQ372157	CgZF3	F-CTTCATCCACTGTTCCTTCTTCCG                         R-GCCTCCTCTTCCTCTTGTTGTTG
CG-J02f_002_E02	FQ327210	CgZF11	F-GCGAAGCAGAAGCAGAAGAAGAG                       R-GGACCACAGACCCAGCATCG
CG-N02f_013_K09	FQ365083	CgZF1	F-AGACCGCTATCTCGGGCCGT                                 R-TGGCGTGAGGGGTGGTTGGT
CGCL2559Contig1	FQ369640,FQ376988	CgERF1	F-GCGGATTCTGCGAAGTTGAGG                                                  R-CGAGGAGTGAGGAGGAGGAAC
CG-N02f_004_I11	FQ366079	CgERF14	F-GGACGAGACCCAGGAAAGACG                                                 R-GCCCACTCCAGCCGCATC
CGCL1993Contig1	FQ325634,FQ375184	CgHD-ZIP2	F-CAACAACGGCGGTGGCAAC                                                            R-CGGCATCCAACCAAAGTAGTAAGG
CG-N02f_038_P11	FQ362432	CgG2-like5	F-CACAGATAGCAGAAGCGTTG                                                         R-AAGCCTCGTTGGTCATAGC
AG-N01f_041_E07	FQ342052	AgARF4	F-TCGGGGTGGAAATTGGTATATGTG                                            R-CTTCGCTCATCTTCTGAACTTCTG
AG-R01f_025_M01	FQ351325	AgbZIP9	F-TCAGGCTCCACACCAACTCAG                                                  R-TTTCGCATTCGGGATCTTCTCG
AG-N01f_038_C02	FQ342334	AgERF8	F-GCTGCTGCGGCTCGGTTC                                                                       R-CGGTGACGGTGACTCCATCG
AG-R01f_023_M04	FQ351523	AgERF20	F-CTCCCAATCCAACAGCAACCC                                                             R-TCAGGAACGGACAGCGAAGG
AG-N01f_037_G22	FQ342384	AgGRAS5	F-GTTCTAGGAAAGTGGAGGTCAAGG                                                      R-CAAGCGGAAGAGGTTACCATAGG
AG-N01f_024_I22	FQ343589	AgWRKY1	F-TATGGACTACAACACCCTCTCAAG                                  R-CACCTGCTGCCTCGTTAGATG
AGCL3603Contig1	FQ332812, FQ338622	AgZF10	F-AACGGGTCAGGCACTTGGG                                 R-TTGGCTTCTTCAGGCGATGTTTC

## Sheet: Feuil2

## Sheet: Feuil3
